# The Current Status of Decompressive Craniectomy in Traumatic Brain Injury

**DOI:** 10.1007/s40719-018-0147-x

**Published:** 2018-09-01

**Authors:** Angelos G. Kolias, Edoardo Viaroli, Andres M. Rubiano, Hadie Adams, Tariq Khan, Deepak Gupta, Amos Adeleye, Corrado Iaccarino, Franco Servadei, Bhagavatula Indira Devi, Peter J. Hutchinson

**Affiliations:** 1 Department of Clinical Neurosciences, Division of Neurosurgery, Addenbrooke’s Hospital & University of Cambridge, Cambridge, CB2 0QQ UK; 2 NIHR Global Health Research Group on Neurotrauma, University of Cambridge, Cambridge, UK; 3 Department of Clinical Neurosciences, Service of Neurosurgery, University Hospital of Lausanne (CHUV), Lausanne, Switzerland; 4 Neuroscience Institute, INUB-MEDITECH Research Group, El Bosque University, Bogotá, Colombia; 5 Department of Neurosurgery, North West General Hospital and Research Center, Peshawar, Pakistan; 6 Department of Neurosurgery, Neurosciences Centre, All India Institute of Medical Sciences, New Delhi, India; 7 Department of Surgery, Division of Neurological Surgery, College of Medicine, University of Ibadan, Ibadan, Nigeria; 8 Department of Neurological Surgery, University College Hospital, Ibadan, Nigeria; 9 Department of Neurosurgery, Azienda Ospedaliero Universitaria di Parma, Parma, Italy; 10 Department of Neurosurgery, Humanitas University and Research Hospital, Milan, Italy; 11 Department of Neurosurgery, National Institute of Mental Health and Neurosciences (NIMHANS), Bangalore, India

**Keywords:** Neurosurgery, Neurotrauma, Decompression, Cranioplasty, Cisternostomy

## Abstract

**Purpose:**

This review describes the evidence base that has helped define the role of decompressive craniectomy (DC) in the management of patients with traumatic brain injury (TBI).

**Recent Findings:**

The publication of two randomized trials (DECRA and RESCUEicp) has strengthened the evidence base. The DECRA trial showed that neuroprotective bifrontal DC for moderate intracranial hypertension is not helpful, whereas the RESCUEicp trial found that last-tier DC for severe and refractory intracranial hypertension can significantly reduce the mortality rate but is associated with a higher rate of disability. These findings have reopened the debate about (1) the indications for DC in various TBI subtypes, (2) alternative techniques (e.g., hinge craniotomy), (3) optimal time and material for cranial reconstruction, and (4) the role of shared decision-making in TBI care. Additionally, the role of primary DC when evacuating an acute subdural hematoma is currently undergoing evaluation in the context of the RESCUE-ASDH randomized trial.

**Summary:**

This review provides an overview of the current evidence base, discusses its limitations, and presents a global perspective on the role of DC, as there is growing recognition that attention should also focus on low- and middle-income countries due to their much greater TBI burden.

## Introduction

Traumatic brain injury (TBI) remains a major public health problem worldwide. It is a leading cause of mortality and disability across the globe, with low- and middle-income countries (LMICs) facing the greatest disease burden [1•]. Brain swelling and intracranial hypertension are well-recognized secondary insults associated with increased mortality and poorer outcomes.

Decompressive craniectomy (DC) refers to the practice of removing a large bone flap and opening the underlying dura in order to control brain swelling and raised intracranial pressure (ICP). This review aims to present the evidence base concerning the role of DC following TBI, to identify areas of uncertainty, and to discuss future directions.

## Historical Perspectives

There is evidence that the practice of trephination (a word coming from Ancient Greek *trypanon*: *to drill*) dates back to around 10,000 B.C. [2]. The first known written report concerning the use of trephination dates back to Ancient Greece, more precisely to Hippocrates, who defined the indications for trephination in relation to different types of skull fractures [2, 3]. The practice of trephination continued during the Roman era, Middle Ages, and Renaissance. One physician whose work was particularly significant was Berengario da Carpi (1466–1530). He provided the first classification of head trauma and its outcome in relation to surgical techniques [4].

In the modern era, Theodor Kocher first described the necessity of opening the skull in the presence of increased intracranial pressure: “if there is no cerebrospinal fluid (CSF) pressure, but brain pressure exists, then pressure relief must be achieved by opening the skull” [5]. The first results of Kocher’s doctrine were published by Harvey Cushing in 1908, who reported a drastic reduction in TBI mortality from 50 to 15% after subtemporal DC [6]. During the twentieth century, different DC techniques were described (hemicraniectomy, circumferential, bifrontal), but lack of consensus about indications and significant variation in outcomes paved the way for randomized trials in the beginning of the twenty-first century [7, 8].

## Definitions

Hemicraniectomy, also known as unilateral DC or frontotemporoparietal craniectomy, refers to the removal of a large frontotemporoparietal bone flap, whereas bifrontal DC refers to the removal of a bone flap extending from the floor of the anterior cranial fossa to the coronal suture, and to the middle cranial fossa floor bilaterally. Wide opening of the dura is a necessary part of the procedure.

## Subtypes of TBI in Which DC Is Used

- A DC is most frequently undertaken in comatose patients with an acute subdural hematoma (ASDH) and associated brain swelling in the early phase after injury [9–11]. In this group of patients, the ASDH is evacuated and a large bone flap is left out either because the brain is bulging beyond the inner table of the skull or because increasing brain swelling (e.g., in a patient with large cerebral contusions) is anticipated in the postoperative period. This type of DC is termed primary and is most frequently a unilateral hemicraniectomy. With regard to timing from injury, a primary DC is usually undertaken within the first 24 h, but in some papers the time window extends to the first 48–72 h [12•, 13].
- A DC can also be undertaken in comatose patients who have parenchymal hemorrhage or contusions (usually frontotemporal) with substantial mass effect. This usually manifests as midline shift (> 5 mm) and/or uncal herniation in the case of predominantly unilateral contusions or obliterated basal cisterns in the case of bilateral contusions. Such patients may initially receive ICP monitoring (if available) and proceed to a DC later if their ICP becomes difficult to control. This type of DC is termed secondary and can be unilateral (for predominantly unilateral pathology) or bifrontal (for predominantly bilateral pathology). Alternatively, patients with severe mass effect and clinical signs of herniation (such as anisocoria) may receive a DC early after injury without prior monitoring of ICP, especially in areas with low availability of neuromonitoring resources [14, 15•].
- Patients who have had a craniotomy (i.e., bone flap replaced at end of procedure) for evacuation of an intracranial hematoma in the early phase after injury occasionally undergo a subsequent DC if their ICP becomes difficult to control or if they deteriorate neurologically with radiological evidence of increasing mass effect. This usually occurs in patients in whom coexisting contusions are blossoming.
- Less frequent indications include closed TBI with diffuse brain swelling without any significant hematomas/contusions, gunshot wound with gross hemorrhage and swelling; and severe blast injury with gross swelling [16–18].

Overall, primary DC at the same time as evacuation of a hematoma is the most frequent scenario for performance of DC [19•]. Secondary DC undertaken after a period of ICP monitoring in order to control refractory elevation of ICP and/or to treat clinical or radiological deterioration is a less frequent indication.

## Strengthening the Evidence Base

In the 1990s, after advances in imaging, prehospital, and intensive care led to improvements in TBI management, the importance of developing the evidence base for DC by conducting robust studies became apparent.

It is widely accepted that well-planned experimental studies can provide robust evidence to inform clinical practice. When one is interested in evaluating the effectiveness of a treatment, a control group, ideally in the context of a randomized controlled trial (RCT), is necessary. Through random assignment of individuals, the treatment and control groups are likely to be balanced in both observable and non-observable characteristics. Consequently, we can be fairly confident that any differences in outcome between the two groups are due to the experimental effect of exposure to the treatment [20].

However, RCTs may not always be feasible for practical or ethical reasons. Additionally, treatments with dramatic effects that are unlikely to have resulted from inadequately controlled biases do not need to be subjected to a RCT. Such an example from the field of TBI would be evacuation of a substantial extradural hematoma in a patient who is neurologically compromised. Furthermore, a number of potential difficulties, such as lack of clinical equipoise, strong patient and clinician preferences, imbalance in surgical expertise, cross-over between groups, and difficulty with blinding, may render surgical trials particularly challenging [21].

New approaches, such as comparative effectiveness research (CER), have become popular in recent years. Non-experimental CER studies—such as the CENTER-TBI project (https://www.center-tbi.eu/)—aim to utilize heterogeneity in care provision and outcomes to compare the effectiveness of treatments that are standard practice in some centers but not in others. Nevertheless, the methodology of non-experimental CER studies and interpretation of their findings remain a work in progress. Therefore, even though these efforts are important and promising, they can only be seen as being complementary to randomized trials. Pragmatic RCTs, which compare two or more treatments in the “real world,” are a form of CER, the so-called experimental CER [22]. Although RCTs and non-experimental CER are both important facets of TBI research, only RCTs are widely accepted as the gold-standard method for assessing the efficacy and effectiveness of therapeutic interventions. Hence, if a question is sufficiently refined to allow the design of an RCT, such a study should be undertaken whenever feasible [23].

## Overview of Main Randomized Trials RCT in TBI

The main randomized trials in the field of DC following TBI are three in number: (1) DECRA trial, which examined the role of neuroprotective, secondary, and bifrontal DC for moderate intracranial hypertension; (2) RESCUEicp trial, which examined the role of last-tier secondary DC for severe and refractory intracranial hypertension; and (3) RESCUE-ASDH trial, which is examining the role of primary DC for ASDH.

The DECRA trial, for which results were published in 2011, enrolled 155 patients with severe diffuse TBI and moderate intracranial hypertension in three different countries (Australia, New Zealand, and Saudi Arabia) [24••]. Patients were eligible for randomization within the first 72 h after trauma if the ICP was higher than 20 mmHg for > 15 min (continuously or intermittently) within a 1-h period and was not responding to first-tier ICP-lowering interventions. Patients were randomized to bifrontal DC or to continuing medical care. The primary endpoint was the extended Glasgow Outcome Scale (GOSE) score at 6 months. The mortality was similar in both groups (19% vs 18%), but more surgical patients had an unfavorable GOSE (70% vs 51%; *p* = 0.02). Following post hoc adjustment for pupil reactivity at baseline, the rate of unfavorable outcome was no longer significantly different between the two arms (adjusted OR 1.90; 95% CI 0.95–3.79). Although DECRA has been widely criticized for various reasons [25], we view it as a valuable trial that addressed a very specific question. On the basis of its findings, we are able to conclude that bifrontal DC should not be used as a neuroprotective measure for moderate posttraumatic intracranial hypertension in well-resourced settings.

The RESCUEicp trial, for which results were published in 2016, enrolled 408 patients with severe and refractory posttraumatic intracranial hypertension in 20 countries [26••]. Patients were eligible for randomization at any time point after trauma if the ICP was higher than 25 mmHg for at least 1 h and did not respond to first-tier and second-tier ICP-lowering interventions. Patients were randomized to secondary DC (bifrontal DC or hemicraniectomy) or standardized medical therapy (with the option of barbiturates after randomization). The primary endpoint was GOSE score at 6 months. DC resulted in substantially lower mortality (26.9% vs 48.9%) but higher rates of vegetative state (8.5% vs 2.1%), lower severe disability (21.9% vs 14.4%), and upper severe disability (independent at home; 15.4% vs 8%) than medical care. The rates of moderate disability and good recovery were similar in the two groups. Nevertheless, surgical patients continued improving beyond the 6 months, and at 12 months, 45.4% of surgical patients had a favorable outcome (upper severe disability or better) compared to 32.4% in the medical group (*p* = 0.01). These results suggest that secondary DC can be helpful as a last-tier intervention to reduce mortality in the subset of TBI patients with severe and refractory posttraumatic intracranial hypertension. However, caution is needed because approximately 40% of extra survivors generated by DC will be dependent on others at 12 months. The contrasting results of DECRA and RESCUEicp arise from differences in study hypotheses, eligibility criteria, and therapeutic protocols; the main differences have been summarized in Table 1 [27].

**Table 1:** Differences between DECRA and RESCUEicp trials

	DECRA	RESCUEicp
Recruitment up to 72 h post-TBI	100% of patients	56% of patients
TBI type	Diffuse injury only	Diffuse injury and/or mass lesions (including contusions and evacuated hematomas)
ICP threshold	> 20 mmHg for 15 min in 1 h	> 25 mmHg for at least 1 h
ICP-lowering therapies before randomization	Tier 1	Tiers 1 and 2
Pooled mortality	18.7%	37.5%
Mortality in DC vs medical group	19 vs 18%	26.9 vs 48.9%
Documented follow-up	6 months	6 and 12 months

The RESCUE-ASDH trial is currently ongoing. This trial aims to address the paucity of high-quality evidence regarding the best surgical strategy (primary DC or craniotomy) for patients with ASDH (Table 2). A previously published survey has shown that a higher percentage of neurosurgeons from other European countries (48/110; 44%) as compared with UK/Irish neurosurgeons (29/138; 21%) use primary DC in more than half of ASDH cases (*p* < 0.001) [28]. A more recent survey of 60 neurosurgeons from the Netherlands and Belgium demonstrated a large variation in the decision to combine ASDH evacuation with a DC [29•]. These results demonstrate that a considerable lack of consensus exists on the indications for primary DC in this context. The RESCUE-ASDH trial was funded by the UK National Institute for Health Research (NIHR) and was launched in 2014 with the aim of comparing primary DC (bone flap left out) with craniotomy (bone flap replaced and fixed) for patients with a serious TBI undergoing evacuation of an ASDH. Similar to real-world practice, eligible patients are randomized intraoperatively after evacuation of their ASDH. Patients with significant brain swelling preventing safe replacement of the bone flap are not suitable for randomization and are being enrolled in a parallel observational cohort. The study is ongoing, and more than 400 patients have been enrolled in the randomized trial from 35 sites worldwide [23].

**Table 2:** Inclusion, exclusion criteria, and outcome measures of RESCUE-ASDH

Inclusion criteria	1. Adult head-injured patients (> 16 years) 2. ASDH on CT 3. The admitting neurosurgeon feels that the hematoma needs to be evacuated with a large bone flap (≥ 11-cm anteroposterior diameter) either by a craniotomy or decompressive craniectomy (patients with additional lesions such as intracerebral hemorrhage/contusions) may be included
Exclusion criteria	1. Bilateral ASDHs both requiring evacuation 2. Previous enrollment in RESCUE-ASDH study 3. Severe pre-existing physical or mental disability or severe co-morbidity which would lead to a poor outcome even if the patient made a full recovery from the head injury
Primary outcome measure	Extended Glasgow Outcome Scale (GOSE) at 12 months
Secondary outcome measures	1. GOSE at 6 months 2. Quality of life (EQ-5D) at discharge from neurosurgical ward, 6 and 12 months 3. Glasgow Coma Scale (GCS) on discharge from the intensive care unit (ICU) and from neurosurgical ward 4. Length of stay in ICU, neurosurgical and rehabilitation unit 5. Discharge destination from neurosurgical ward 6. Mortality 7. Serious adverse events and surgical complications 8. Subsequent readmissions within the 1-year follow-up period 9. Return to operating theater for cranial surgery within 2 weeks after randomization 10. Hydrocephalus requiring shunt insertion within the 12 months follow-up period 11. Therapy intensity level in the ICU 12. Economic evaluation

Finally, another study that should be mentioned is a randomized trial that took place in five centers in China to compare outcomes after a standard-sized trauma DC (12 × 15-cm flap) vs a limited DC (6 × 8-cm flap) in severe TBI patients with refractory intracranial hypertension [30•]. The authors recruited 486 patients in total and found that the mortality rate was lower (26% vs 35%) and favorable outcome rate higher (39.8% vs 28.6%) after standard trauma DC compared to limited DC (*p* < 0.05).

## Limitations of Current Evidence Base

About 90% of worldwide trauma-related deaths occur in LMICs. However, less than 10% of the RESCUEicp patient population was enrolled in LMICs (36/408 patients from six countries), whereas all patients in the DECRA study were from high-income countries (HICs). This fact raises some important questions. Firstly, is it possible to extrapolate the results from studies taking place in HICs (where prehospital, acute neurosurgical, and postacute care are generally delivered in a more systematic way) to the results that can be expected in LMICs? Secondly, is it possible for neurosurgeons working in LMICs to follow recommendations derived from the DECRA and RESCUEicp studies, given that ICP monitoring may not be available in their daily practices? Nevertheless, the burden of TBI is much higher in LMICs, and patients are being treated for TBI despite the absence of evidence directly applicable to these countries. These are issues that are receiving further study as part of efforts to improve global neurotrauma care. Such an initiative is the NIHR Global Health Research Group on Neurotrauma, which is supported by the World Federation of Neurosurgical Societies. This international group aims to improve neurotrauma care in LMICs.

Additionally, more attention needs to be paid to the issue of cranial reconstruction (cranioplasty) following DC. Neurological dysfunction associated with large skull defects has been proposed as an important factor that can influence the outcome of patients after DC [31•, 32]. Small, uncontrolled studies suggest that earlier cranioplasty (within three months of DC) may facilitate rehabilitation and may even independently improve long-term outcome [33••]. From the surgical viewpoint, the tissue planes seem to be more favorable when a cranioplasty is undertaken early. However, due to a longstanding belief that the rate of infection may be higher with earlier cranioplasty, many of these operations are undertaken in a delayed fashion. This is clearly an important area for future research. Additionally, despite the plethora of different materials available for cranioplasty following DC (e.g., titanium, PEEK, hydroxyapatite, and the patient’s own bone flap), uncertainty remains as to whether anyone of them is associated with better outcomes [34]. Cranioplasty-related costs differ among various materials, which is an important consideration for LMICs as well as several HICs with state-funded healthcare systems.

Another area of debate is the use of floating or hinged bone flaps as a potential decompressive method for TBI. Floating or hinged bone flaps have the potential to control at least moderate swelling while at the same time obviating the need for a subsequent cranioplasty [35, 36•]. This is an important consideration in resource-limited settings. These techniques could be evaluated prospectively in randomized controlled studies, as if they are proven to be beneficial, they could advance the care of patients in LMICs.

The opening of basal cisterns (cisternostomy) has also been suggested as a surgical maneuver for managing posttraumatic brain swelling and elevated ICP [37]. However, this technique requires a microscope and instruments for microneurosurgery, which may limit its utility in resource-constrained settings. Additionally, the fact that an external cisternal drain is left in situ (with CSF drainage of 150–200 ml/day for 6 days in a recent case report) [38] suggests that the therapeutic effect of cisternostomy may be mediated at least in part by CSF drainage. A much simpler and faster method for achieving CSF drainage is an external ventricular drain (ventriculostomy), but due to the additional hypothesized benefits of cisternostomy, it has become clear that the only way to determine the utility of cisternostomy is through the conduct of randomized trials. Despite the criticism that is often directed to randomized surgical trials, they remain the optimal study design for rigorous evaluation of surgical interventions, techniques, and devices [39, 40].

Finally, it should be emphasized that the perspectives of patients and their families should always be considered when determining the degree of “acceptable” disability. Additionally, the degree of “acceptable” disability varies from person to person and is dependent on many factors, such as culture, social environment, and religion. Therefore, the indirect input of the patient (as best as is possible) and of families is very important when determining the degree of acceptable disability for an individual, and consequently whether a DC should be considered [41, 42].

## Conclusions

Several TBI subtypes associated with brain swelling and/or raised ICP can be managed by DC. However, current evidence from multicenter clinical trials suggests that (1) early neuroprotective bifrontal DC for mild to moderate intracranial hypertension is not superior to medical management for patients with diffuse TBI, and (2) unilateral or bifrontal DC used as a last-tier therapy for patients with severe, sustained, and refractory posttraumatic intracranial hypertension leads to a substantial mortality reduction but increases disability (both lower and upper severe disability) compared to medical management. The RESCUE-ASDH trial is currently open and aims to define the role of primary DC for patients with acute subdural hematomas and swelling. The global neurosurgical community needs to consider the roles of DC, cranioplasty, and other decompressive procedures (such as floating or hinge craniotomy) not just in HICs but also, and perhaps more importantly, in LMICs due to their much greater TBI burden.
